# Risk factors for acute kidney injury and mortality in high risk patients undergoing cardiac surgery

**DOI:** 10.1371/journal.pone.0252209

**Published:** 2021-05-21

**Authors:** Giuseppe Filiberto Serraino, Michele Provenzano, Federica Jiritano, Ashour Michael, Nicola Ielapi, Pasquale Mastroroberto, Michele Andreucci, Raffaele Serra

**Affiliations:** 1 Department of Experimental and Clinical Medicine, University of Catanzaro, Catanzaro, Italy; 2 Department of Medical and Surgical Sciences, University of Catanzaro, Catanzaro, Italy; 3 Department of Health Sciences, University Magna Graecia of Catanzaro, Catanzaro, Italy; 4 “Sapienza” University of Rome, Department of Public Health and Infectious Disease, Roma, Italy; 5 Interuniversity Center of Phlebolymphology (CIFL), International Research and Educational Program in Clinical and Experimental Biotechnology, University Magna Graecia of Catanzaro, Catanzaro, Italy; IRCCS Policlinico S.Donato, ITALY

## Abstract

**Background:**

Acute Kidney Injury (AKI) represents a clinical condition with poor prognosis. The incidence of AKI in hospitalized patients was about 22–57%. Patients undergoing cardiac surgery (CS) are particularly exposed to AKI because of the related oxidative stress, inflammation and ischemia-reperfusion damage. Hence, the risk profile of patients undergoing CS who develop AKI and who are consequently at increased mortality risk deserves further investigation.

**Methods:**

We designed a retrospective study examining consecutive patients undergoing any type of open-heart surgery from January to December 2018. Patients with a history of AKI were excluded. AKI was diagnosed according to KDIGO criteria. Univariate associations between clinical variables and AKI were tested using logistic regression analysis. Variable thresholds maximizing the association with AKI were measured with the Youden index. Multivariable logistic regression analysis was performed to assess predictors of AKI through backward selection. Mortality risk factors were assessed through the Cox proportional hazard model.

**Results:**

We studied 158 patients (mean age 51.2±9.7 years) of which 74.7% were males. Types of procedures performed were: isolated coronary artery bypass (CABG, 50.6%), valve (28.5%), aortic (3.2%) and combined (17.7%) surgery. Overall, incidence of AKI was 34.2%. At multivariable analysis, young age (*p* = 0.016), low blood glucose levels (*p* = 0.028), estimated Glomerular Filtration Rate (*p* = 0.007), pH (*p* = 0.008), type of intervention (*p* = 0.031), prolonged extracorporeal circulation (ECC, *p* = 0.028) and cross-clamp (*p* = 0.021) times were associated with AKI. The threshold for detecting AKI were 91 and 51 minutes for ECC and cross-clamp times, respectively. At survival analysis, the presence of AKI, prolonged ECC and cross-clamp times, and low blood glucose levels forecasted mortality.

**Conclusions:**

AKI is common among CS patients and associates with shortened life-expectancy. Several pre-operative and intra-operative predictors are associated with AKI and future mortality. Future studies, aiming at improving prognosis in high-risk patients, by a stricter control of these factors, are awaited.

## Introduction

Acute Kidney Injury (AKI) represents a severe clinical condition characterized by an increased risk of mortality, particularly in hospitalized patients [1]. The global incidence of AKI in hospital settings, according to *Kidney Disease*: *Improving Global Outcomes (KDIGO)* definitions is about 22% - 57%, with an increasing prevalence due to the concomitant increasing trend in cardiovascular disease comorbidity [2, 3]. The onset of AKI is common among patients undergoing cardiac surgery (CS) because of a series of intrinsic factors, including the transfusion of large volumes of exogenous blood products, extracorporeal circulation techniques, high doses of vasopressors, which, all together, determine cycles of ischemia-reperfusion, oxidative damage, and inflammation [4, 5]. The incidence of AKI after CS is related to the type of operative procedure [6]. In a large cohort study, enrolling more than 3.000 patients who underwent CS and without a previous history of kidney disease, the rate of AKI was higher (reaching up to 59% patients) in aortic surgery, including aortic interventions combined with other interventions, as compared to isolated coronary artery bypass surgery (CABG) (37%), valve surgery (49%) and thoracic surgery (33%) [7]. Moreover, patients who developed AKI were also at significant increased risk of long-term mortality if they underwent CABG, aortic and thoracic surgery, but not valve surgery [7]. However, this topic is still controversial. Another observational analysis, indeed, found no difference in 3-month mortality risk among patients with AKI, who underwent different types of Cardiac Surgery [8]. Owing to these controversies, the knowledge of risk factors for AKI remains a crucial research endeavor, since it could help clinicians in improving prevention, a prompt diagnosis and management of AKI in this setting of patients. Several risk scores for AKI have already been published [9, 10]. Among them, the ‘any-stage AKI risk score’ has shown a good performance for all stages of AKI and is also available in clinical practice as a web-based calculator [10]. Nevertheless, the inclusion of novel risk factors of AKI in a risk prognostic model has been widely prompted, also on the basis of the results of several clinical trials that have failed in demonstrating the efficacy of prevention strategies, such as the use of statins at high-doses or the remote ischemic preconditioning (RIPC) treatment [4, 11, 12]. We designed a retrospective, observational, study enrolling patients who underwent CS to evaluate both the predictors of the onset of AKI and the role of cardiorenal markers on individual prognosis (i.e. mortality endpoint) over time.

## Methods

### Study design and procedures

This is a retrospective cohort study examining 160 consecutively patients referred to our CS Unit at *Magna Graecia* University of Catanzaro (Italy) from January 1^st^ to December 1^st^, 2018. The study was approved by the Institutional Review Board (IRB) of CIFL -Interuniversity Center of Phlebolymphology- at *Magna Graecia* University of Catanzaro (Id approval number: ER.ALL.2018.37A). All the individual data collected were fully anonymized before the analysis. Patients were enrolled according to the following criteria: age > 18 years, undergoing CS with extracorporeal circulation (ECC). Patients requiring emergency operation, with a preoperative infective status or with a history of AKI during the previous 3-months, or renal replacement therapy were excluded. AKI was defined according to the KDIGO classification, based on the change in serum creatinine levels (≥ 0.3 mg/dL within 48 hours or ≥ 50% within 7 days) or urine output volume less than 0.5 mL/kg/hour for >6 hours [13]. Surgical procedures were realized through median sternotomy. In CABG, the left internal mammary artery (LIMA) was harvested in a pedicled fashion and anastomosed to the left anterior descending coronary artery (LAD). When harvested, a pedicled radial artery (RA) was used as Y-graft with LIMA. Single or sequential saphenous vein grafting was left to the surgeon’s discretion. Valvular procedures included aortic (AVR) and mitral (MVR) valve replacement. An age of 65 years was used as the threshold for choosing mechanical or biological prosthesis after counseling with the patient. Surgical procedures across the aorta included prosthetic replacement of ascending aorta in patients with aneurysmatic dilation. Aortic clamping was performed in all operations. The use of ECC was standardized. Total ECC flow was maintained at 2.6 L/min/m^2^ in all operations. Systemic temperature was kept at 34°C. Myocardial protection was always achieved with intermittent antegrade and retrograde hyperkalemic blood cardioplegia [14–18].

Preoperative data about comorbidities, blood pressure, body mass index (BMI) and therapy were collected. Laboratory values, echocardiographic measures and arterial blood gas (ABG) were obtained at 5 different times: upon hospital admission, which was considered the baseline study visit; at 1h, 24h and 48h after the operation; at discharge. For hemoglobin (hgb) <7.5 g/dL (or hematocrit [Hct] <22 percent), initial treatment during CPB is removal of fluid by hemoconcentration when possible. Transfusion of packed red blood cells (RBCs) is reasonable if Hgb remains <7.5 g/dL when ultrafiltration is not possible or is ineffective [19–24]. The presence of hypoglycemia, as a categorical variable, was defined by the presence of blood glucose < 75 mg/dL. Postoperative data regarding the main clinical parameters and medications were also collected. After the hospital discharge, patients were followed for monitoring complications and treatment with periodical in-hospital visits with a frequency variable based on the severity of their clinical parameters. Follow-up lasted until August 31^st^ 2020, the onset of death or the last visit to the CS Unit.

### Statistical analysis

Continuous variables were reported as mean ± standard deviation (SD) or median and interquartile range (IQR) based on their distribution. Comparison between groups was assessed by unpaired t-test or Mann-Whitney test. Categorical variables were analyzed using the Chi-square test. Univariate association between the main clinical variables and the onset of AKI was assessed by means of logistic regression analysis. To find a cut-point that maximizes the variable’s ability to differentiate AKI from no-AKI endpoint the Youden index (J) was computed. Next, a backward variable selection method, with an elimination criterion of p<0.10, was performed to fit the multivariable logistic regression model. Multicollinearity was assessed with variance inflation factors (VIF), with values greater than 10 considered as cause for concern. First-order interactions between covariates included in the models have been tested. For the survival analysis, multivariable Cox proportional hazard regression analysis was used by assessing the effect of clinical or laboratory parameters on the onset of mortality over time. Data was analyzed using STATA 14 (StataCorp. College Station, TX, USA).

## Results

Two out 160 patients were excluded from the initial cohort because of in-hospital death. The overall cohort (Table 1) was characterized by a high-risk profile as testified by the high frequency of diabetes (40.5%) and arterial hypertension (79.1%) as well as by a high BMI (27.4±4.3 kg/m^2^).

**Table 1:** Basal characteristics of patients by acute kidney injury (AKI) status.

	Overall (n = 158)	No-AKI (n = 104)	AKI (n = 54)	*p*
Age, *years*	51.2±9.7	52.3±10.4	48.9±8.1	0.040
Male gender, *%*	74.7	77.9	68.5	0.199
Diabetes, *%*	40.5	43.3	35.2	0.326
Hypertension, *%*	79.1	76.0	85.2	0.176
Body Mass Index, *kg/m*^*2*^	27.4±4.3	27.2±4.3	27.8±4.4	0.472
Intervention type, *%*				0.014
Isolated CABG	50.6	57.7	37.1	
Others (including valve surgery, combined and aortic surgery)	49.4	42.3	63.0	
Atrial fibrillation, %	18.4	17.3	20.4	0.637
EURO Score	7.2	7.1	7.3	0.747
Blood Pressure, *mmHg*	127±18/71±11	126±19/71±11	129±21/71±13	0.378/0.940
eGFR, *mL/min/1*.*73 m*^*2*^	72.3±23.0	76.8±21.4	63.5±23.5	<0.001
Hemoglobin, *g/dL*	12.7±1.9	12.8±1.9	12.4±1.9	0.252
Urea, *mg/dL*	45 [37–61]	43 [36–57]	49 [40–68]	0.015
Glycemia, *mg/dL*	121±48	126±52	112±37	0.083
Myoglobin, μg/L	36 [24–53]	35 [23–52]	38 [27–61]	0.239
Troponin, *mg/L*	0.02 [0.01–0.06]	0.02 [0.01–0.10]	0.02 [0.01–0.04]	0.260
Creatine phosphokinase, *IU/L*	51 [35–78]	52 [36–78]	49 [34–78]	0.508
Lactate dehydrogenase, *U/L*	351 [300–410]	353 [301–415]	348 [299–405]	0.630
Pro-BNP, *pg/mL*	508 [239–1848]	461 [239–757]	1271 [271–2812]	0.154
PTT, *seconds*	35 [31–46]	37 [32–47]	34 [30–41]	0.062
Pulmonary artery pressure, *mmHg*	40±13	38±11	42±16	0.218
Blood pH, *pH scale*	7.36±0.07	7.36±0.06	7.34±0.08	0.079
Sodium, *mEq/L*	133±6	134±6	131±6	0.061
Time of ECC, *min*	121±46	115±44	132±48	0.036
Time of Clamp, *min*	78±34	74±33	86±36	0.053
Length of stay, *days*	18 [14–23]	17 [14–21]	20 [16–34]	0.001
Diuretics	22.8	19.2	29.6	0.139
RAASi, *% pts*	32.3	31.7	33.3	0.838
Statins, *% pts*	21.5	19.2	25.9	0.331

In the overall cohort, isolated CABG was the most performed operation (Table 2).

**Table 2:** Distribution of operative procedures in the whole population.

Type of Surgical Procedure	Frequency N (%)
Isolated coronary artery bypass surgery	80 (50.6)
Valve surgery	45 (28.5)
Aortic surgery	5 (3.2)
Combined interventions^a^	28 (17.7)

^a^coronary artery bypass surgery + valve and/or aortic interventions.

Furthermore, in the whole cohort, AKI occurred in 54 out 158 patients (34.2%). AKI group was characterized by a statistically significant younger age (*p* = 0.040), a lower pre-operative eGFR (*p*<0.001), and higher extracorporeal circulation (ECC) and cross-clamp times (*p* = 0.036 and 0.053, respectively). Frequency of isolated CABG was higher in the non-AKI group, whereas other operative procedures were more prevalent in the AKI group (*p* = 0.014). The onset of AKI was also associated with an increased length of hospitalization, being of 3 days in median higher as compared with no-AKI group (*p* = 0.001). In the post-operative period, 53.2% patients were started with furosemide, 36.7% with amiodarone, 44.9% with norepinephrine and 5.1% with epinephrine treatment. Differences for these drugs frequencies between AKI and non-AKI groups were significant for furosemide (70.4% in AKI vs. 44.2% in non-AKI, p = 0.002), norepinephrine (59.3% in AKI vs. 37.5% in non-AKI group, p = 0.009) and epinephrine (11.1% in AKI vs. 1.9% in non-AKI, p = 0.012). Treatment with antibiotics was needed in 17.1% patients with no differences between AKI and non-AKI groups. After intervention, 116 patients (73.4%) received blood transfusion, being this more frequent in AKI group (87.0% vs. 66.4%, p = 0.005), 37 patients (23.4%) needed intra-aortic balloon pump (IABP), 12 (7.6%) developed sepsis and 21 (13.2%) developed atrial fibrillation (FA). Differences of IABP, sepsis and FA between AKI and non-AKI group were not significant.

From univariate logistic analysis (Table 3), a backward selection analysis was applied.

**Table 3:** Univariate logistic regression analysis for acute kidney injury (AKI) in patients undergoing cardiac surgery.

Variables	Coefficient (95%CI)	*p*
Age, *years*	-0.038 (-0.075- -0.001)	0.043
Intervention type, *others vs*. *isolated CABG*	0.841 (0.165–1.516)	0.015
Length of stay, *days*	0.024 (0.001–0.047)	0.041
eGFR, *mL/min/1*.*73 m*^*2*^	-0.026 (-0.041- -0.010)	0.001
Urea, *mg/dL*	0.008 (-0.001–0.018)	0.095
Glycemia, *mg/dL*	-0.007 (-0.015–0.001)	0.088
Myoglobin^a^, μg/L	0.429 (-0.071–0.929)	0.093
Troponin^a^, *mg/L*	-0.231 (-0.487–0.025)	0.077
Blood pH, *pH scale*	-4.465 (-9.491–0.561)	0.082
Sodium, *mEq/L*	-0.052 (-0.107–0.003)	0.064
Time of ECC, *min*	0.076 (0.003–0.150)	0.041
Time of Clamp, *min*	0.096 (-0.003–0.195)	0.057

^a^variables were log-transformed due to the skewed distribution.

The resulting multivariable logistic analysis, depicted in Table 4 showed that young age, lower blood glucose levels and eGFR, and pH reduction were associated with an increased risk for AKI. Similarly, prolonged ECC and cross-clamp times were found to be significant risk factors for AKI. Risk increased every 10 minutes of prolonged ECC (9%) and cross-clamp (13%) times.

**Table 4:** Multivariable logistic regression analysis for determinants of acute kidney injury (AKI).

Characteristics	Odds Ratios (95%CI)	*p*
Age, *years*	0.94 (0.90–0.99)	0.016
Intervention type, *others vs*. *isolated CABG*	2.19 (1.07–4.47)	0.031
Glycemia, *mg/dL*	0.99 (0.98–0.99)	0.028
eGFR, *mL/min/1*.*73 m*^*2*^	0.97 (0.96–0.99)	0.007
Blood pH, *1 unit decrease*	2.20 (1.22–3.97)	0.008
Time of ECC^a^, 10 *min*	1.09 (1.01–1.18)	0.028
Time of Clamp^a^, 10 *min*	1.13 (1.02–1.26)	0.021

^a^time of clamp and time of ECC have been added alternatively to the model due to multicollinearity (Variance Inflation Factor = 24.48). ECC, Extracorporeal Circulation; eGFR, estimated Glomerular Filtration Rate; CABG, Coronary Artery Bypass Surgery.

When Youden indexes have been computed, a value of 91 minutes (Sensitivity = 83% and Specificity = 78%) was found as the threshold of ECC that discriminates the risk for AKI, whereas the better discrimination for cross-clamp time was found above 51 minutes (Sensitivity = 85% and Specificity = 81%). When blood glucose was added as a categorical variable, the presence of hypoglycemia remained a strong and significant predictor of AKI (OR 2.55, 95% CI: 1.06–6.15). ECC and cross-clamp times have been separately added to the multivariable logistic regression due to collinearity (VIF = 24.48). Risk for AKI also varied by the type of operative procedure with an odds ratio (OR) of 2.19 for patients who underwent valve, aortic and combined surgery versus isolated CABG. Interaction between ECC time and type of intervention (β = 0.36, p = 0.001) and between cross-clamp time and type of intervention (β = 0.54, p<0.001) were significant.

**Fig 1:**
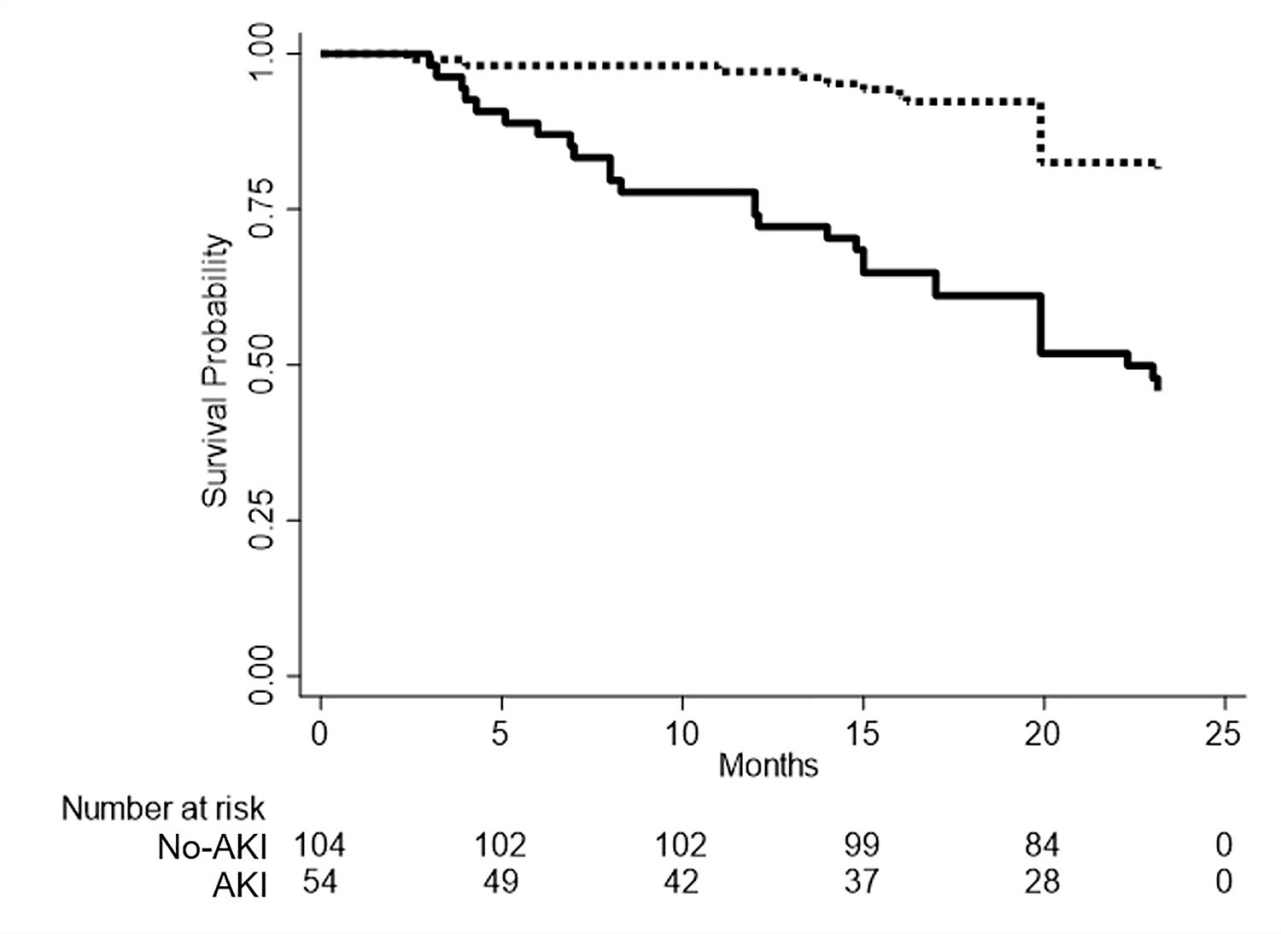
Survival probability (Kaplan-Meier estimates) of patients undergoing cardiac surgery classified by acute kidney injury (AKI) condition. Red line represents patients with AKI, whereas navy line refers to patients without AKI.

During a median follow-up of 20.3 months, 48 all-cause death events were recorded. Of this, 30 patients (62.5%) died for CV fatal events, 10 (20.8%) for complications after hemodialysis and 8 (16.7%) for other causes. Crude risk of death was significantly higher in AKI vs. non-AKI patients (log-rank test *p*<0.001, Fig 1) with a 3.69-fold higher annual rate of mortality in AKI group.

**Fig 2:**
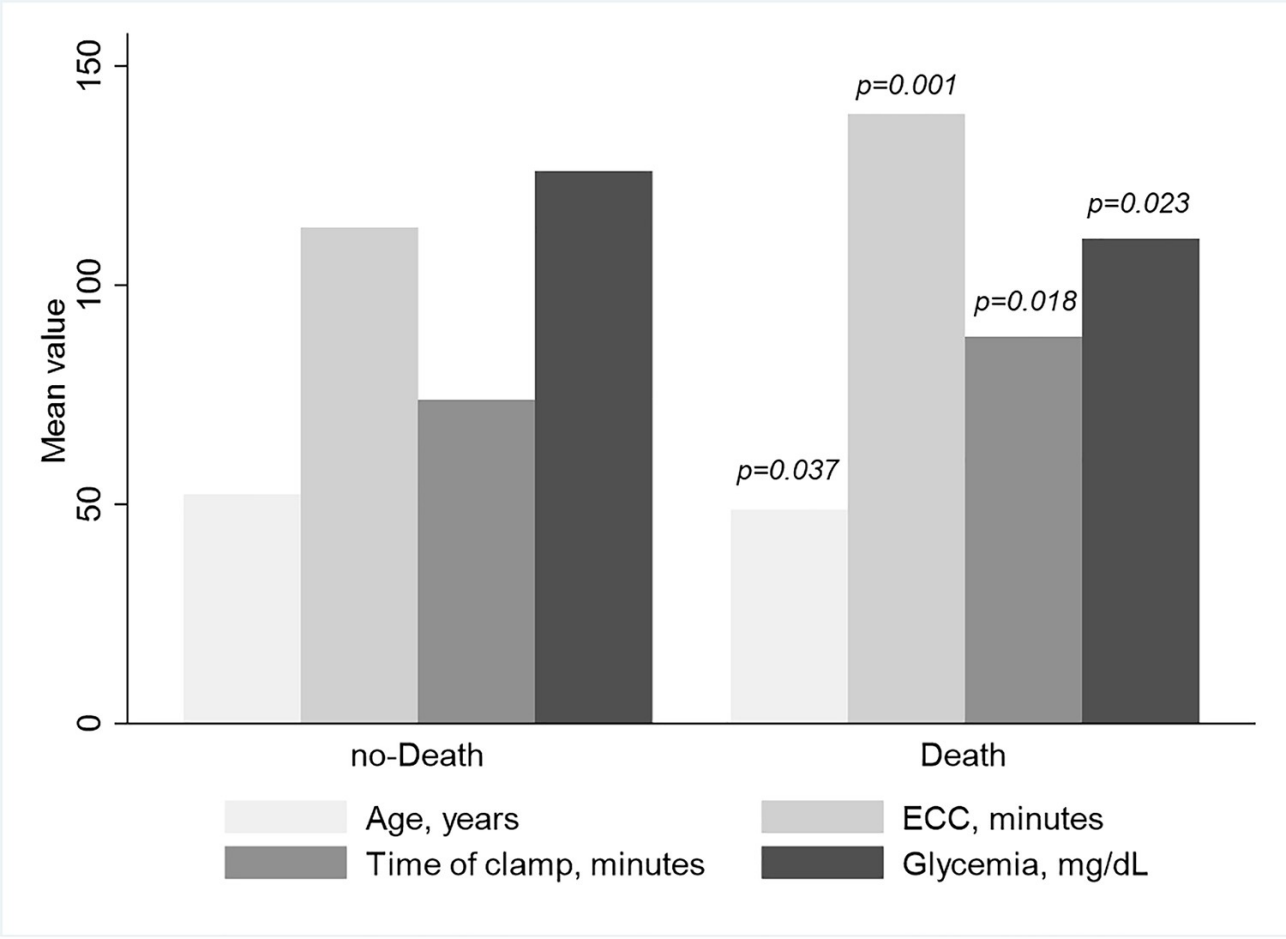
Mean distribution of patients’ clinical parameters according to their mortality status.

At multivariable Cox-proportional hazard model, adjusted for the covariates which differed as averaged values between death vs. no-death group (Fig 2 and Table 5) and the presence/absence of AKI, lower blood glucose levels, ECC and cross-clamp times, and the presence of AKI were significant predictors for mortality endpoint.

**Table 5:** Mortality risk by baseline patients’ characteristics.

	HR	95% CI	*p*
Age, *years*	0.97	0.94–1.01	0.099
AKI, *yes vs*. *no*	3.14	1.73–5.72	<0.001
Glycemia, *mg/dL*	0.99	0.98–0.99	0.031
Time of ECC^a^, 10 *min*	1.08	1.02–1.14	0.011
Time of Clamp^a^, 10 *min*	1.09	1.01–1.18	0.046

^a^variables alternatively added to the model due to the collinearity and to avoid the model overfitting.

For sensitivity analysis, we replaced blood glucose level as a continuous variable with the presence/absence of hypoglycemia which was associated with an increased risk for all-cause mortality (HR 2.45, 95% CI: 1.35–4.43).

## Discussion

Risk stratification for the cardiac surgical patient represents an important tool that physicians may use for pre-operative decision-making and intra-operative management. In most cases, several comorbidities often affect this cohort of patients worsening the outcomes [7, 8]. This evidence is confirmed in our cohort in which about 41% of patients suffered from type-2 diabetes, 79% from hypertension, and all patients (100%) were affected by previous cardiovascular disease. In addition, more than 30% of patients developed AKI or died during follow-up. It has been widely demonstrated that cardiovascular disease is strictly linked to kidney impairment, since an acute or chronic disorder in one organ may induce an acute or chronic disorder in another one [25, 26]. The presence of cardiac dysfunction leads to the activation of the renin-angiotensin-aldosterone-system (RAAS), sympathetic nervous system and vasopressin secretion, leading to fluid retention [27]. Moreover, CS procedures contribute *per se* to trigger AKI and, consequently to increase the individuals’ risk [28]. The previous risk scores showed that a large number of preoperative, intraoperative and post-operative risk factors can predict the onset of AKI in patients undergoing CS [4, 9, 10, 29, 30]. However, these scores were not largely used in clinical practice, so that the scientific community prompted to implement studies exploring the risk factors of AKI and prognosis in high-risk patients, those who would most benefit from novel treatments and prevention strategies [31]. The methodological novelty of our study is the inclusion of both preoperative and intraoperative factors at the same time for evaluating the risk for AKI and subsequent mortality, with an adequate sample size. As the main results of this study, we confirmed the importance of well-established risk factors of AKI, such as the preoperative levels of eGFR, time of ECC and aortic cross-clamp times and expanded this association to other potential, still less explored, risk factors, such as the type of surgical procedure, blood glucose and blood pH. Furthermore, we showed that the risk factors for AKI may also predict a poor prognosis, besides and beyond the presence of AKI itself.

With respect to AKI, ECC and cross-clamp times are two peculiar characteristics of CS that could elicit AKI [32]. Extracorporeal circulation contributes to AKI because of red blood cell hemolysis and the systemic inflammatory response syndrome (SIRS), that induces the increase in blood levels of mediators, such as IL-6, IL-8 and TNF-α [32–34]. A crucial point that influences these pathways is represented by the ECC duration. We observed that mean ECC time was slightly higher compared with that reported in other studies enrolling patients undergoing no-valve interventions, but it was comparable with the one measured in more complex surgical procedures [35, 36]. However, those studies that evaluated ECC duration in both valve and no-valve intervention were limited by small sample sizes with a consequent weak power for statistical analysis [35, 36]. We found that 91 minutes of ECC was found as the threshold above which the risk for AKI started to increase significantly. This evidence is higher when compared to an ECC time longer than 70 minutes reported by other studies [35–37]. Cross-clamp time is another risk factor for renal dysfunction since it associates with ischemic-reperfusion damage. Cross-clamp time observed in our cohort was similar to that reported in other studies [35, 36]. We found that 51 minutes of cross-clamp time was the best value which discriminates patients with and without AKI. The importance of ECC and cross-clamp times is even reinforced by the significant association of both parameters with an increased risk for mortality in our patients. We observed that patients, who had undergone valve, aortic and combined interventions, had about a 2-fold increased risk for AKI compared with those receiving isolated CABG. We could explain this finding by considering that isolated CABG is usually associated with lower ECC and cross-clamp times [38]. Moreover, patients undergoing procedures other than CABG are, *per se*, at higher basal risk. Interestingly, we found an interaction between ECC time and type of intervention (p = 0.001) as well as between cross-clamp time and type of intervention (p<0.001) with the onset of AKI. This may be explained with the higher ECC and cross-clamp times (132±48 minutes and 101±32 minutes, respectively) in patients with other procedures when compared to isolated CABG (115±44 minutes and 56±17 minutes, respectively). With respect to preoperative risk factors, we found that baseline eGFR was a strong determinant for the onset of AKI. This has been previously shown and has been recognized as one of the stronger predictors of AKI [7, 34]. Originally, we found that young age and lower blood glucose levels can represent independent risk factors for AKI, regardless of the baseline levels of kidney function. With respect to the association between young age and AKI, this piece of data is apparently in contrast to previous literature reports which show how older age is associated with a raised risk of AKI [29, 30]. However, we could explain this finding taking into account the inverse association between age and the systemic inflammatory response syndrome (SIRS) to the ECC. Previous studies have shown that the mean age of patients with SIRS is significantly lower than the one of those without SIRS [39, 40]. Hence, younger patients seem to require a strong monitoring of renal function to the same extent as in older patients, because the risk for AKI remains non-trivial. Regarding glycemia, we may argue that almost all existing risk prediction models, evaluating the preoperative risk factors for AKI, are focused on the presence of diabetes as a determinant of AKI [9, 10, 29, 30]. Based on our results, we could not exclude that also the opposite clinical condition, namely lower blood glucose levels, may be a predisposing factor for AKI. Indeed, evidence for an association between hypoglycemia and AKI have been recently shown, particularly in hospitalized patients [41]. Kidneys are directly involved in glucose metabolism, with the renal cortex being responsible for up to 30% of total gluconeogenesis. Hospitalization *per se* could enhance mechanisms that lead to hypoglycemia, such as inadequate nutrition intake, lack of counter regulatory mechanisms and reduction of kidney function [7, 32, 42, 43]. Furthermore, we excluded that the association between AKI and blood glucose levels may be dependent of insulin treatment. In fact, frequency of insulin use before intervention did not differ (0 = 0.623) between patients who developed AKI and those who did not. We found that the presence of AKI itself is associated with an increased mortality risk over time and that this is partially explained by the complexity of surgical procedures as testified by the association of time of ECC and cross-clamp time with mortality rate. Interestingly, we found new evidence of association between AKI and mortality, by reporting an extended follow-up, reaching more than 20 months. This confirms the importance for clinicians to monitor kidney parameters which play a crucial prognostic role in high-risk patients [43–47].

Moreover, reduction in arterial pH levels are pivotal to diagnose acidosis, which is the most frequent in patients affected by renal impairment [48]. Metabolic acidosis is a predictor of AKI and deserves a strict monitoring since this parameter, if associated with the presence of hyperkalemia, oliguria, uremia and/or volume overload, represents an indicator for renal replacement therapy [49].

### Strengths and limitations

The single-center dimension of the present study limits the generalizability of the obtained results. Moreover, the small sample size did not allow to adjust for all the potential confounders of the association between CS and AKI. However, the literature lacks any strong evidence on this topic. The few papers investigating intraoperative variables studied more limited cohorts with a weaker statistical analysis compared with the present paper [36, 37]. We did not collect glycated hemoglobin (HbA1c) values in our patients that would be useful to further investigate the association between blood glucose levels and AKI. However, future study may give more insight around the hypothesis we have generated.

In conclusion, the onset of AKI in the CS setting still remains a relevant epidemiologic and clinical problem. In fact, the development of AKI is associated with a worse prognosis over time. We may assert that a predictive model, which encompasses pre-operative and intraoperative risk factors, provides a more comprehensive risk profile of patients at increased risk for AKI and subsequent mortality. Patients undergoing complex interventions, low blood glucose levels, acidosis, preexistent eGFR reduction, and long ECC and cross-clamp times have been found to have an increased risk for AKI. Further attention must be paid to younger patients too, who may present a more severe inflammatory response to surgical procedures. Moreover, patients with AKI, low blood glucose, ECC and cross-clamp duration are at increased mortality risk during the follow-up. Further investigations should clarify whether a better control of these risk factors would ultimately result in a protection against AKI and a better prognosis.

## Supporting information

S1 Database (SAV)
